# Daptomycin-induced Acute Eosinophilic Pneumonia

**DOI:** 10.7759/cureus.2899

**Published:** 2018-06-30

**Authors:** Sundeep Kumar, Israel Acosta-Sanchez, Natarajan Rajagopalan

**Affiliations:** 1 Internal Medicine, University of Central Florida College of Medicine, Orlando, USA; 2 Pulmonary and Critical Care, Orlando VA Medical Center , Orlando, USA

**Keywords:** acute eosinophilic pneumonia, daptomycin induced acute eosinophilic pneumonia, acute respiratory failure, pulmonary eosinophilia, adverse drug effects, daptomycin adverse effects

## Abstract

Acute eosinophilic pneumonia (AEP) is a rare entity, often resulting in respiratory failure and the attended mortality. Daptomycin-induced AEP results from immune-mediated pulmonary epithelial cell injury. A 65-year-old male on treatment with intravenous daptomycin for three weeks came to the hospital for worsening dyspnea and acute hypoxemic respiratory failure. Computerized tomography (CT) of the chest was done, revealing bilateral pulmonary infiltrates. He underwent bronchoscopy that showed predominant pulmonary eosinophilia. The bacterial, fungal, viral, and mycobacterial cultures were all negative. Daptomycin was discontinued, and the patient was started on steroid therapy. He received a two-week course of steroids with a rapid taper, attaining complete recovery with a near-complete resolution of pulmonary infiltrates. A shorter course of steroid therapy should be sufficient to treat a case, as indicated in our case. Commonly used diagnostic criteria for AEP using more than 25% of pulmonary eosinophilia should be tailored to patient-related factors.

## Introduction

Acute eosinophilic pneumonia (AEP) is a rare disorder, often resulting in acute respiratory failure with an attended mortality. AEP is characterized by the infiltration of pulmonary parenchyma with eosinophils and is often associated with peripheral eosinophilia [1-2]. AEP has been associated with antibiotics, certain chemicals, and non-steroidal anti-inflammatory agents [3]. The pathophysiology of AEP involves the detection of the offending agent by alveolar macrophages. The macrophages present the antigen to T helper cells, which, in turn, produce interleukin-5. Interleukin-5 along with eotaxin is produced by endothelial cells. Epithelial cells, macrophages, and airway smooth muscle cells result in the accumulation of eosinophils in the alveolar spaces. This inflammatory cascade results in acute epithelial injury [4]. Daptomycin-related AEP remains a diagnostic and therapeutic challenge. We present a patient with AEP following therapy with daptomycin.

## Case presentation

A 65-year-old gentleman with past medical history significant for chronic kidney disease stage 3, liver cirrhosis, and thoracolumbar spinal stenosis presented to the hospital because of progressive dyspnea, fever, and non-productive cough for two days. He underwent laminectomy for spinal stenosis, later complicated by T5-8 vertebral osteomyelitis with epidural phlegmon, requiring drainage and debridement with hardware removal. He was started on empiric antibiotics with intravenous vancomycin and cefepime, which were replaced with daptomycin when phlegmon cultures grew methicillin-sensitive Staphylococcus aureus. He received daptomycin for three weeks.

**Figure 1:**
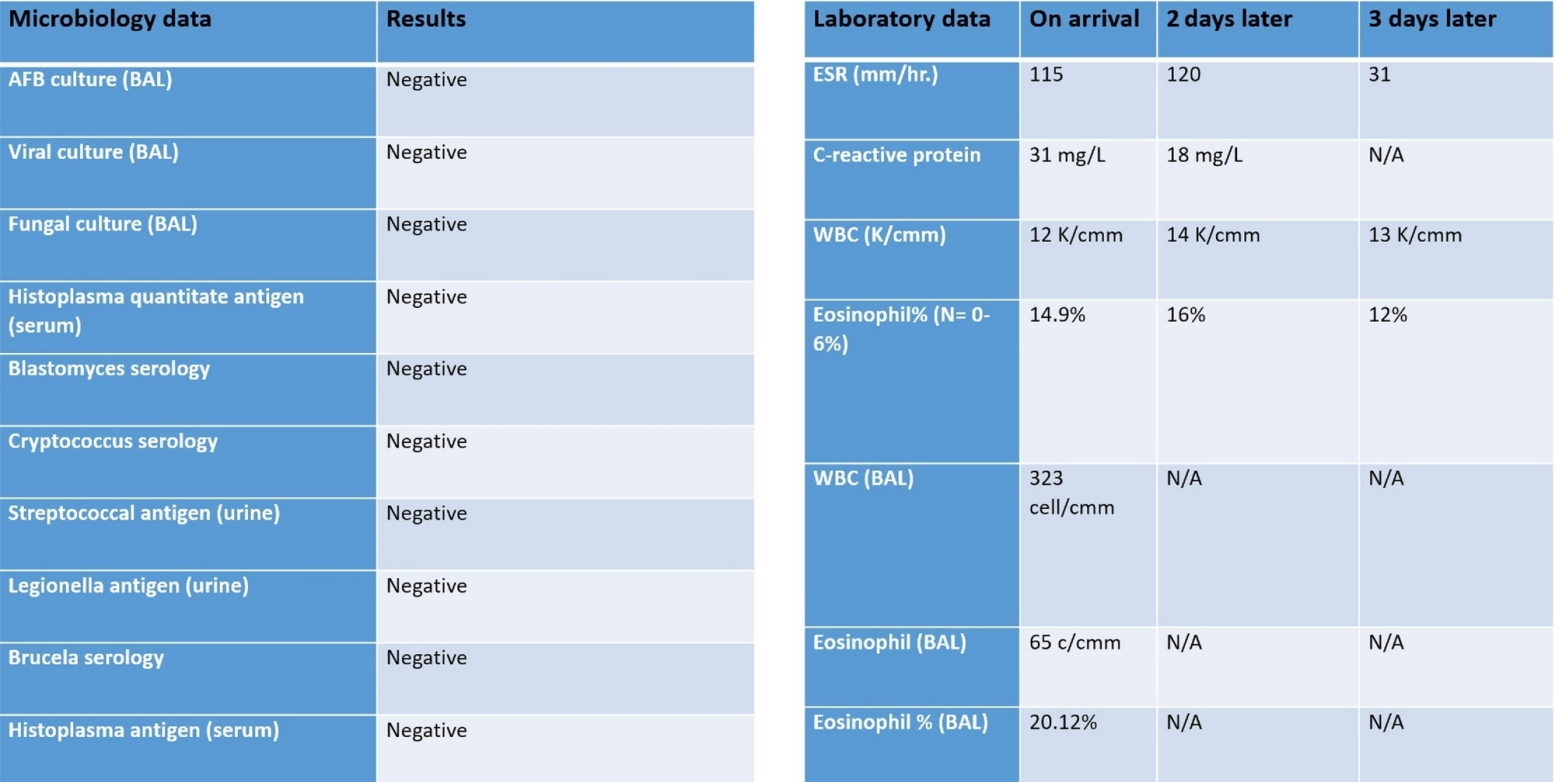
Microbiology and laboratory investigations AFB: acid-fast bacilli; BAL: bronchoalveolar lavage; ESR: erythrocyte sedimentation rate; WBC: white blood cell

**Figure 2:**
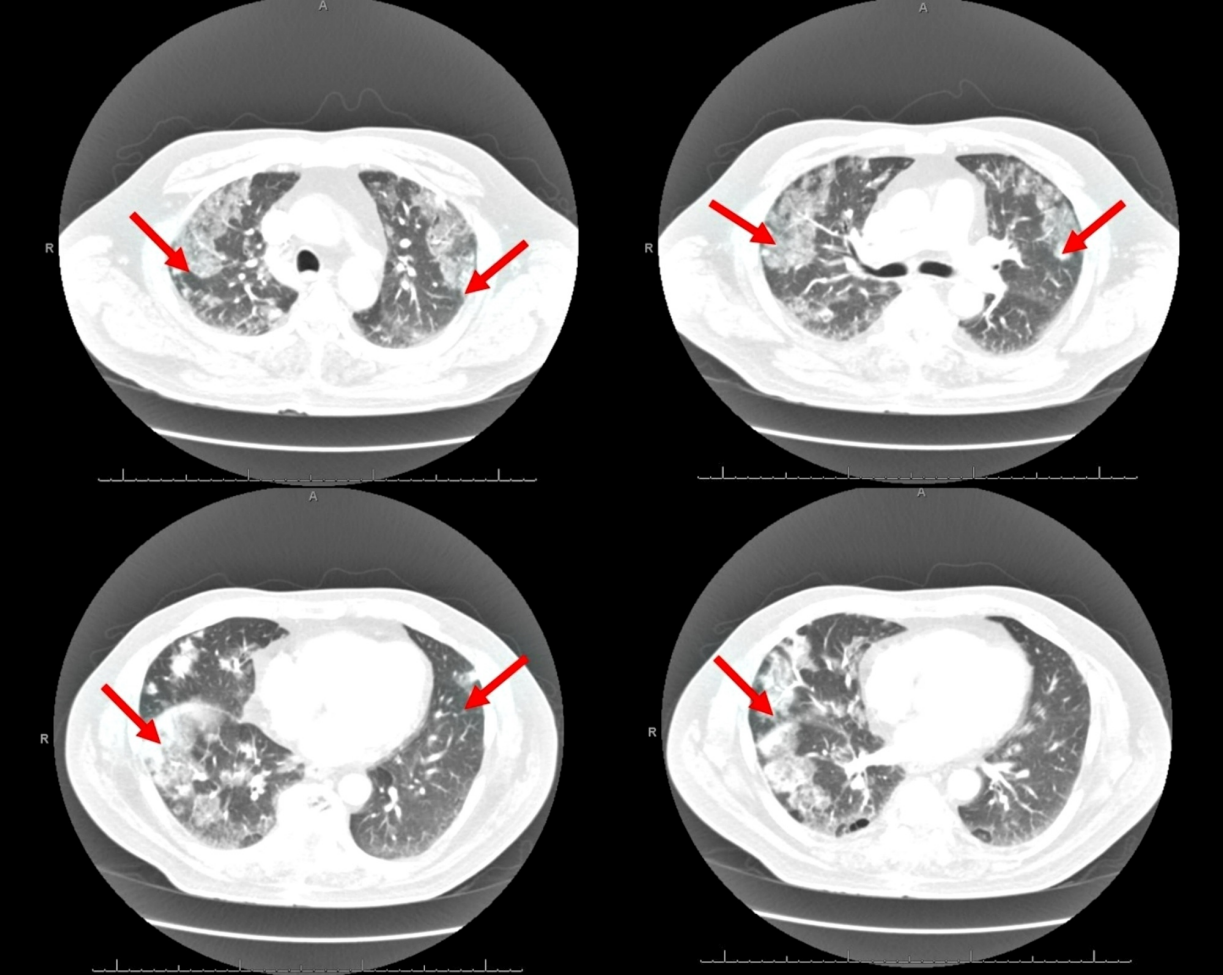
Computerized tomography (CT) scan of chest at presentation showing bilateral pulmonary infiltrates as indicated by red arrows

**Figure 3:**
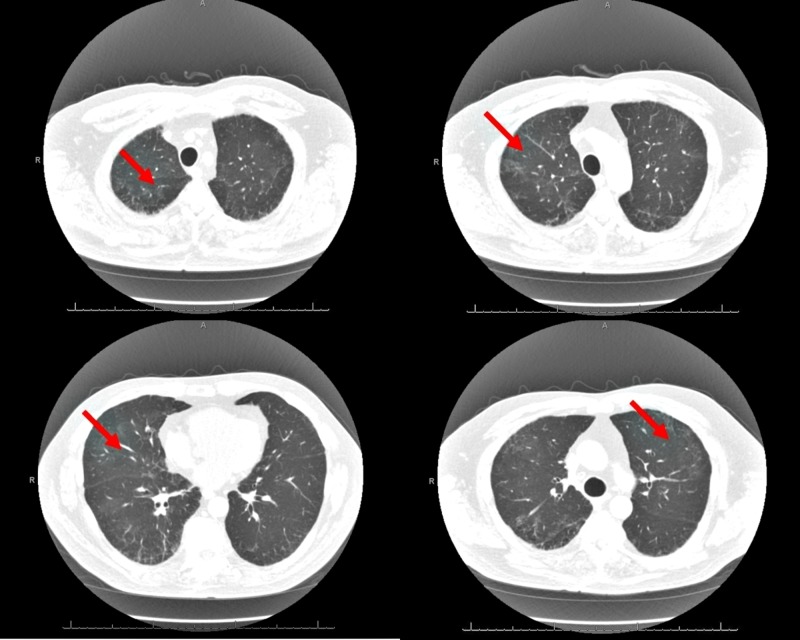
Computerized tomography (CT) scan of chest at follow-up visit three weeks post-treatment showing resolution of pulmonary infiltrates as indicated by red arrows

He appeared in respiratory distress with tachypnea and was hypoxemic on arrival to the hospital with a peripheral capillary oxygen saturation (SpO2) of 90%. Pulmonary examination revealed diffuse scattered bi-basal crackles. Laboratory studies revealed a polymorphonuclear leukocytosis and eosinophilia. Pulmonary infection from other infective agents, including bacterial, fungal, mycobacterial, and viral organisms, was considered in this patient and appropriate culture and serologies were sent but reported negative. The remainder of the laboratory and microbiology data are shown in Figure 1. His computerized tomography (CT) chest scan with contrast at arrival revealed bilateral pulmonary infiltrates, as shown in Figure 2. A diagnosis of daptomycin-induced AEP was strongly suspected. Bronchoscopy and bronchoalveolar lavage (BAL) were performed the next day, which revealed an eosinophil count of >20% in lavage. He was started on intravenous solumedrol and daptomycin was discontinued. He responded to this regimen in the next 24-48 hours with subjective improvement and reduced oxygen requirements. He was discharged home on a reducing course of steroids for two weeks. A repeat CT chest scan at his three-week follow-up revealed a resolution of the pulmonary infiltrates (Figure 3).

## Discussion

Acute eosinophilic pneumonia in relation to daptomycin has been characterized into definite, probable, possible, and unlikely [1]. To date, only a limited number of cases of daptomycin-induced AEP have been described. After a post-marketing survey, the product labeling was updated to include AEP and pulmonary eosinophilia among the adverse effects. The prosed pathophysiology involves the presentation of the drug or a drug-hapten combination by the macrophages to the T-helper cell results in interleukin–5 release; this, along with macrophage secreted eotaxin, results in eosinophilic migration to the lungs. The precise mechanism for daptomycin-induced lung injury is unknown but is believed to be related to daptomycin binding to pulmonary surfactant culminating in epithelial injury [2].

Daptomycin-induced AEP is seen in the elderly, receiving doses in the range of 4-10 mg/kg/day. Renal failure is associated with an increased risk of developing AEP. Peripheral eosinophilia is present in the majority of cases [3]. A diagnosis of AEP does not require a lung biopsy and can be made on clinical criteria. However, a biopsy is helpful in uncertain diagnosis or in non-responders to conventional therapy [5-6].

Acute eosinophilic pneumonia is characterized by the presence of fever, dyspnea, non-productive cough with diffuse pulmonary infiltrates, frequently leading to hypoxemic respiratory failure [4]. Currently, the following criteria are used for the diagnosis of daptomycin-induced eosinophilic pneumonia: (1) recent daptomycin exposure, (2) fever, (3) dyspnea with hypoxemic respiratory failure, (4) new infiltrates on chest radiography, (5) BAL with > 25% eosinophils, and (6) clinical improvement following daptomycin withdrawal. The presence of eosinophils in BAL represents an interplay between the host immune system and immunogen. People with lowered immune defenses may not mount an adequate cellular response. In the proper clinical setting suggestive of AEP secondary to daptomycin, the arbitrary number of 25% may not be necessary.

This case also enlightens the importance of timely recognition and the treatment of daptomycin-induced AEP. We recommend performing bronchoscopy and BAL even though the history, clinical, and radiological findings are highly suggestive of AEP. From the literature, it is still not clear as to how long steroids should be given. The relapse of AEP on the premature discontinuation of steroids has rarely been reported. In patients with underlying infection, there is a concern regarding the exacerbation of infection with steroid use. In our opinion, a shorter course of steroid with a rapid taper should be sufficient to treat the majority of cases, in addition to discontinuing daptomycin. In our patient, prednisone was given for two weeks and no further relapse of his pulmonary symptoms at follow-up. Healthcare providers should be aware of this complication. The prompt discontinuation of daptomycin and a short course of steroids should prevent respiratory failure and reduce morbidity and mortality.

## Conclusions

Daptomycin-induced AEP is increasingly seen in patients receiving the high doses of daptomycin, often resulting in respiratory failure and the attendant mortality. Bronchoscopy and BAL should be considered in all cases suspected of AEP. A shorter course of steroids with a rapid taper should be sufficient to treat the majority of cases. A lung biopsy is only required in cases with a questionable diagnosis or treatment failures. Commonly used diagnostic criteria should be revised to address patient-related factors.
